# Short-Term Outcomes Analysis Comparing Open, Laparoscopic, Laparoscopic-Assisted, and Robotic Distal Gastrectomy for Locally Advanced Gastric Cancer: A Randomized Trials Network Analysis

**DOI:** 10.3390/cancers16091620

**Published:** 2024-04-23

**Authors:** Michele Manara, Alberto Aiolfi, Andrea Sozzi, Matteo Calì, Federica Grasso, Emanuele Rausa, Gianluca Bonitta, Luigi Bonavina, Davide Bona

**Affiliations:** 1 I.R.C.C.S. Ospedale Galeazzi–Sant’Ambrogio, Division of General Surgery, Department of Biomedical Science for Health, University of Milan, Via C. Belgioioso, 173, 20157 Milan, Italy; michele.mnra@gmail.com (M.M.); davide.bona@unimi.it (D.B.); 2 Department of Biomedical Sciences for Health, Division of General and Foregut Surgery, IRCCS Policlinico San Donato, University of Milan, 20097 Milan, Italy; luigi.bonavina@unimi.it

**Keywords:** distal gastric cancer, open distal gastrectomy, laparoscopic-assisted distal gastrectomy, robotic distal gastrectomy, Bayesian network meta-analysis

## Abstract

**Simple Summary:**

The role of minimally invasive surgery for the treatment of locally advanced gastric cancer (AGC) remains controversial. The present network meta-analysis demonstrates that short-term outcomes for open (Op-DG), totally laparoscopic (Lap-DG), laparoscopic-assisted (LapAs-DG), and robotic distal gastrectomy (Rob-DG) seem comparable. Similarly, the total number of retrieved lymph nodes and resection margin appear equivalent. LapAs-DG, Lap-DG, and Rob-DG performed in referral centers by dedicated surgeons have comparable short-term outcomes to Op-DG for locally AGC.

**Abstract:**

Background. Minimally invasive surgery for the treatment of locally advanced gastric cancer (AGC) is debated. The aim of this study was to execute a comprehensive assessment of principal surgical treatments for resectable distal gastric cancer. Methods. Systematic review and randomized controlled trials (RCTs) network meta-analysis. Open (Op-DG), laparoscopic-assisted (LapAs-DG), totally laparoscopic (Lap-DG), and robotic distal gastrectomy (Rob-DG) were compared. Pooled effect-size measures were the risk ratio (RR), the weighted mean difference (WMD), and the 95% credible intervals (CrIs). Results. Ten RCTs (3823 patients) were included. Overall, 1012 (26.5%) underwent Lap-DG, 902 (23.6%) LapAs-DG, 1768 (46.2%) Op-DG, and 141 (3.7%) Rob-DG. Anastomotic leak, severe complications (Clavien–Dindo > 3), and in-hospital mortality were comparable. No differences were observed for reoperation rate, pulmonary complications, postoperative bleeding requiring transfusion, surgical-site infection, cardiovascular complications, number of harvested lymph nodes, and tumor-free resection margins. Compared to Op-DG, Lap-DG and LapAs-DG showed a significantly reduced intraoperative blood loss with a trend toward shorter time to first flatus and reduced length of stay. Conclusions. LapAs-DG, Lap-DG, and Rob-DG performed in referral centers by dedicated surgeons have comparable short-term outcomes to Op-DG for locally AGC.

## 1. Introduction

Gastric cancer is the fifth most diagnosed cancer and the fifth leading cause of death worldwide [1]. Open distal gastrectomy (Op-DG) with D2 lymphadenectomy represents the backbone of curative intent in resectable locally advanced gastric cancer (AGC) located in the distal stomach/antrum [2,3,4]. The first laparoscopic-assisted distal gastrectomy (LapAs-DG) was described by Kitano et al. in 1991 in a patient with pre-pyloric early gastric cancer (EGC) [5]. Because of the promising results, LapAs-DG has become globally accepted in the surgical community [6,7,8,9]. In parallel, the development of innovative laparoscopic technologies and revolutionary robotic platforms introduced new surgical facilities for the completion of safe intracorporeal anastomosis and gastrointestinal tract reconstruction [10,11,12,13]. Therefore, Ballesta et al., in 1996, described the first totally laparoscopic distal gastrectomy (Lap-DG) for distal gastric cancer with intracorporeal anastomosis [14]. Ultimately, during the early 2000s, robotic distal gastrectomy (Rob-DG) was highlighted for its undeniable benefits, including enhanced ergonomics, advanced imaging capabilities, and increased maneuverability facilitated by endo-wristed instruments, leading to improved technical precision [15,16].

Because of the growing surgical expertise, the utilization of minimally invasive techniques extended from EGC to locally AGC. However, controversies exist concerning indications, postoperative outcomes, long-term survival, and cancer recurrence [17,18,19,20,21,22]. Previously published retrospective studies and pairwise meta-analyses compared the safety and effectiveness of LapAs-DG vs. Op-DG and Lap-DG vs. Op-DG [23,24,25]. However, the results were biased and heterogeneous because of the study design and demographic imbalances. Recent pairwise meta-analyses attempted to moderate these limitations by including high-quality observational studies and randomized controlled trials (RCTs); however, results were limited by simple pairwise analysis [26,27]. Because of the publication of recent RCTs reporting data on locally AGC, additive evidence has become available [28,29,30,31,32,33,34,35,36,37].

Hence, the intent of this study was to perform a comprehensive RCTs network meta-analysis to examine short-term outcomes among Op-DG, LapAs-DG, Lap-DG, and Rob-DG in patients with locally AGC.

## 2. Materials and Methods

A systematic literature review up to 31 January 2024 was executed according to the preferred items for systematic reviews and network meta-analyses checklist (PRISMA-NMA) guidelines [38]. PubMed, MEDLINE, Web of Science, Embase, Scopus, and Cochrane Central Library were inquired with a combination of the following Mesh terms: (((gastric) OR (stomach)) AND ((cancer) OR (tumor))) AND ((open distal gastrectomy) OR (laparoscopic distal gastrectomy) OR (laparoscopic-assisted distal gastrectomy) OR (robotic distal gastrectomy)) AND ((locally advanced)) AND ((anastomotic leak) OR (mortality) OR (complication*) OR (short-term)) [39]. No additional ethical approval was required since the present analysis was based on a previously published series. PROSPERO registration number CRD42024505588. 

### 2.1. Eligibility Criteria

Inclusion criteria were as follows: (1) RCTs comparing Op-DG, Rob-DG, Lap-DG, LapAs-DG for the treatment of gastric cancer located in the distal stomach/antrum; (2) RCTs reporting short-term outcomes measurements (at least one of the a priori-defined primary outcomes); (3) studies focusing mainly on locally AGC; and (4) among the studies based on the same group of patients, the ones reporting short-term outcomes were included. Exclusion criteria were as follows: (1) non-comparative studies; (2) articles not written in English; (3) studies reporting mixed data, including other surgical procedures; and (4) studies focusing on or mainly reporting data for EGC.

### 2.2. Selection Process

Three authors (M.M., M.C., and A.S.) conducted independent literature reviews according to the aforementioned eligibility criteria. All titles were screened, and suitable articles’ abstracts were evaluated more extensively for inclusion. The reference lists of all eligible articles were carefully reviewed for further studies. After the removal of duplicates, conflicts were settled by two additional blinded reviewers (A.A. and D.B.).

### 2.3. Data Collection Process

Data were extracted by two authors (M.M., A.A.) and compared at the end of the data collection period. Any discrepancies were dealt with by a third author (D.B.). The following data were collected: authors, study period, country, number of patients, age, sex, body mass index (BMI), surgical approach, reconstruction strategies, intraoperative data (operating time, intraoperative blood loss, conversion rate, number of lymph nodes retrieved), tumor characteristics (pathological tumor stage, histology, size, residual tumor classification), postoperative data (time to first flatus, time to first oral intake, time to first ambulation), overall postoperative complications, severe postoperative complications (Clavien–Dindo ≥ 3), reoperation, anastomotic leak, postoperative bleeding, pancreatic injury/leak, surgical-site infection (SSI), cardiovascular complications, pulmonary complications, hospital length of stay, and mortality.

### 2.4. Outcomes of Interest and Definitions

Primary outcomes were anastomotic leak, severe complications, and in-hospital mortality. Severe complication was defined as greater than or equal to 3 according to the Clavien–Dindo classification of postoperative complications [40]. Secondary outcomes were operative time (minutes), intraoperative blood loss (mL), number of retrieved lymph nodes, time to first flatus (days), time to first oral intake (days), time to first ambulation (days), overall complications, reoperation, postoperative bleeding requiring transfusion, pancreatic injury, pancreatic leak, SSI, pulmonary complications, cardiovascular complications, and hospital length of stay (HLOS) (days). Lap-DG was defined as totally minimally invasive DG with intracorporeal anastomosis completion and alimentary tract reconstruction. LapAs-DG was defined in the case of extracorporeal anastomosis and alimentary tract completion [41].

### 2.5. Quality Assessment

The Cochrane risk-of-bias tool was used to assess the methodological quality of the identified RCTs [42]. Two authors independently assessed the methodological quality of the selected trials according to the following: (1) method of randomization, (2) allocation concealment, (3) baseline comparability of study groups, and (4) blinding and completeness of follow-up. Trials were then graded as having low (green circle), high (red circle), or unclear (yellow circle) risk of bias.

### 2.6. Statistical Analyses

A comprehensive Bayesian network meta-analysis was conducted [43,44], employing the risk ratio (RR) for categorical outcomes and the weighted mean difference (WMD) for continuous outcomes. For RR analysis, a “skeptical” prior distribution with mean and scale set to 0 and 0.4, respectively, was utilized and integrated into a conventional consistency binomial/log model. To account for heterogeneity, an informative half-normal prior with a mean of zero and a scale of 0.5 was applied across treatment comparisons. Sensitivity analysis regarding the choice of the prior distribution for the between-study variability parameter was undertaken [45]. Statistical heterogeneity (measured by the I^2^ index) was categorized as low (≤25%), moderate (50–75%), or high (≥75%) [46]. Inference was computed using mean and relative 95% credible intervals (CrIs) based on Markov chain Monte Carlo (MCMC) with 300,000 iterations following a burn-in period of 30,000 iterations. A parameter was deemed statistically significant if its 95% CrI excluded the null-hypothesis value [47]. Leverage values plotted against the square root of the residual deviance were employed to identify potential outliers. The transitivity assumption was assessed, and descriptive statistics were generated to compare baseline participant characteristics across studies and treatment comparisons. Confidence in estimates of the outcome was evaluated using confidence in network meta-analysis (CINeMA) [48]. Statistical analyses were conducted with JAGS 4.3.2 and R-Cran 3.4 [49,50,51].

## 3. Results

### 3.1. Systematic Review

**Figure 1:**
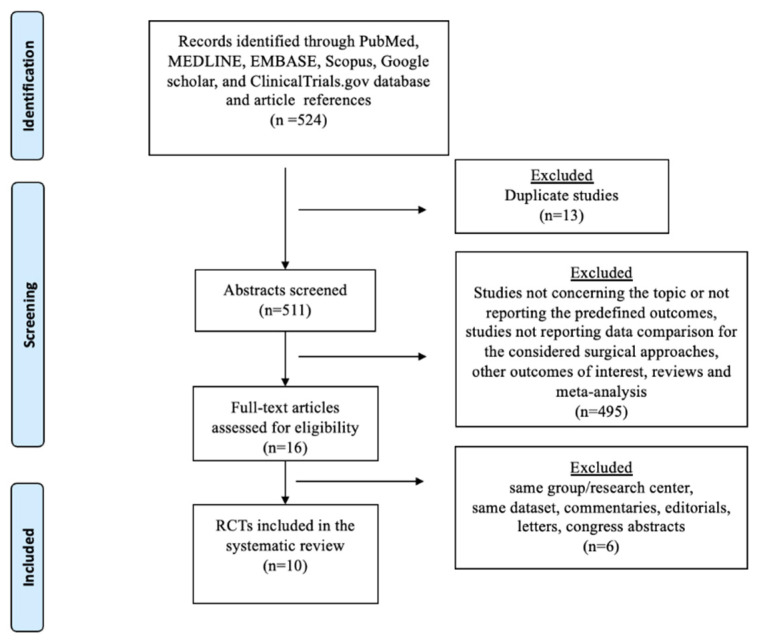
The preferred reporting items for systematic reviews and network meta-analyses (PRISMA-NMA) flowchart.

The database search retrieved 524 publications. The PRISMA flowchart for the literature search is shown in Figure 1. Among screened publications, 10 RCTs met the inclusion and exclusion criteria and were considered in the qualitative analysis. The quality of the included studies is summarized in Supplementary Figure S1. The randomization method was specified in eight studies, and the operating surgeon’s proficiency and surgical quality control were specified in nine and seven studies, respectively. Surgeon and patient blinding were reported in one study, whereas seven studies reported a dedicated powered analysis (Supplementary Table S1).

Overall, 3823 patients were included in the quantitative analysis. Demographic data are summarized in Table 1. Among them, 1012 (26.5%) underwent Lap-DG, 902 (23.6%) LapAs-DG, 1768 (46.2%) Op-DG, and 141 (3.7%) Rob-DG. The majority were males (68.4%), the age of the patient population ranged from 26 to 81 years, while preoperative BMI ranged from 19.4 to 26.7 kg/m^2^. The most common tumor histology was intestinal or differentiated (66.3%) and diffused or undifferentiated (33.7%). Pathologic tumor staging was detailed in nine studies: 34% were stage I tumors, 27.7% were stage II, 36.6% were stage III, and 1.7% were stage IV. Overall, 95 patients (2.5%) underwent neoadjuvant treatments, while adjuvant treatment was reported only in two studies, with 132 patients (34.9%) treated [24,34]. There were no apparent violations of the transitivity assumption. This conclusion was drawn from several factors: consistency of the common treatment (Op-DG) across trials, uniform distribution of effect modifiers across studies, and the potential for participants to be randomized to any treatment compared in the analysis. Additionally, the design-by-treatment interaction model indicated no significant inconsistency. Descriptive statistics are reported in Table 2.

### 3.2. Primary Outcomes

**Figure 2:**
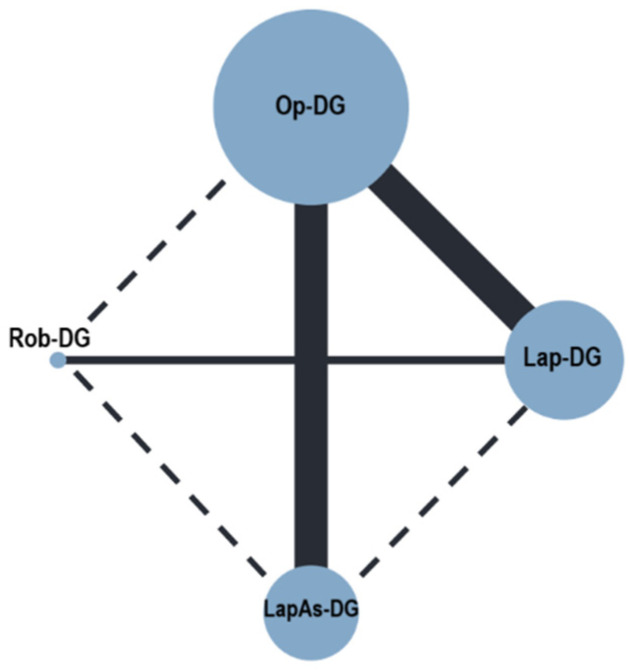
Network geometry for severe postoperative complications (Clavien–Dindo III–IV). Node sizes reflect the sample size, while edge widths reflect the number of studies for a specific pairwise comparison.

**Figure 3:**
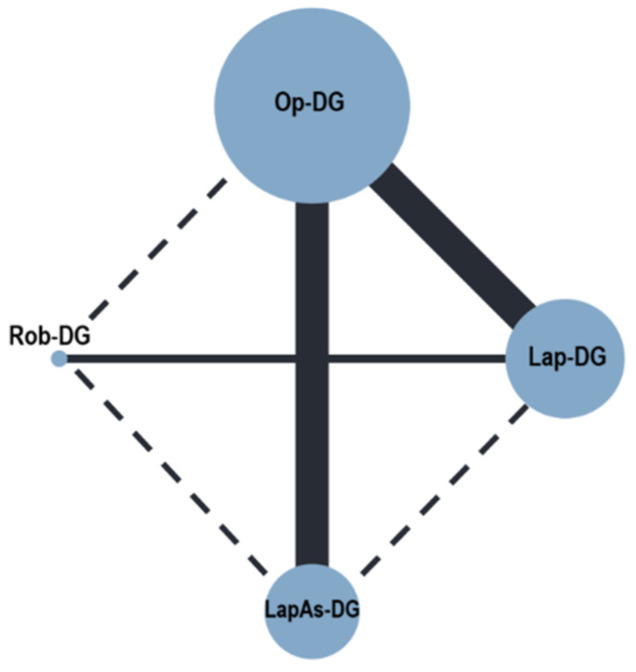
Network geometry for in-hospital mortality. Node sizes reflect the sample size, while edge widths reflect the number of studies for a specific pairwise comparison.

**Figure 4:**
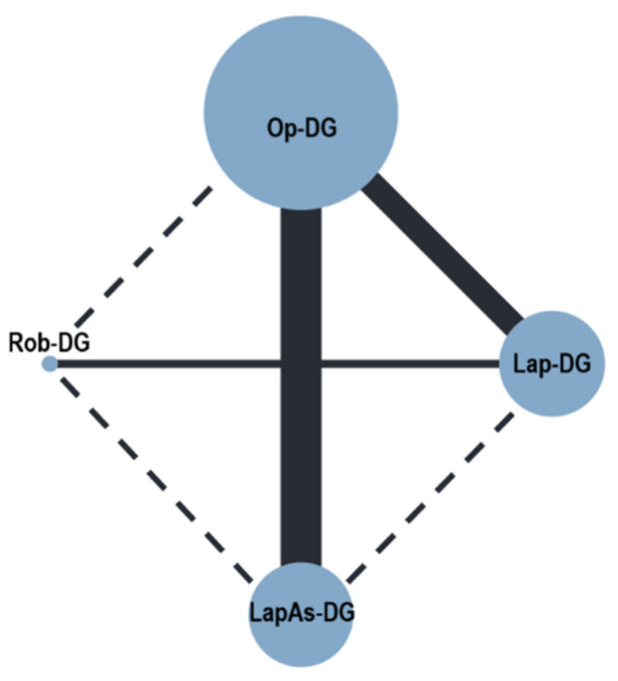
Network geometry for postoperative anastomotic leak. Node sizes reflect the sample size, while edge widths reflect the number of studies for a specific pairwise comparison.

Severe postoperative complications (Figure 2) were reported in nine studies (3627 patients) [28,29,30,31,32,33,34,35,36]. No differences were observed for Lap-DG vs. Op-DG (RR = 1.17; 95% CrI 0.69–1.99), LapAs-DG vs. Op-DG (RR = 1.82; 95% CrI 0.91–3.07), and Rob-DG vs. Op-DG (RR = 0.81; 95% CrI 0.34–2.14). In-hospital mortality (Figure 3) was reported in nine studies (3627 patients) [28,29,30,31,32,33,34,35,36]; no significant differences were observed for Lap-DG versus Op-DG (RR = 1.25; 95% CrI 0.58–2.67), LapAs-DG versus Op-DG (RR = 1.46; 95% CrI 0.67–3.17), and Rob-DG versus Op-DG (RR = 1.13; 95% CrI 0.38–3.30). Anastomotic leak rate (Figure 4) was reported in nine studies (3627 patients) [28,29,30,32,33,34,35,36,37]; no significant differences were observed for Lap-DG versus Op-DG (RR = 1.24; 95% CrI 0.58–2.61), LapAs-DG versus Op-DG (RR = 1.58; 95% CrI 0.72–3.39), and Rob-DG versus Op-DG (RR = 1.06; 95% CrI 0.37–3.06). The treatment ranking evaluation classified Rob-DG as the surgical approach with the lowest probability of being ranked as the first treatment for severe complications (19.8%), anastomotic leak (29%), and in-hospital mortality (28.2%).

### 3.3. Secondary Outcomes

Operative time (10 studies, 3823 patients) [28,29,30,31,32,33,34,35,36,37] was significantly shorter for Op-DG vs. Lap-DG (WMD = 64.17; 95% CrI −75.04; −53.32), LapAs-DG (WMD = 39.26; 95% CrI −49.21; −29.32), and Rob-DG (WMD = 83.83; 95% CrI −107.3; −60.23). LapAs-DG was associated with a significantly shorter operative time compared to Lap-DG (WMD = 24.91; 95% CrI −39.62; −10.17) and Rob-DG (WMD = 44.56; 95% CrI −70.13; −18.93). No significant difference was reported for Lap-DG vs. Rob-DG (WMD = 19.64; 95% CrI −40.57; 1.28). Intraoperative blood loss (nine studies, 3627 patients) [28,29,30,32,33,34,35,36,37] was significantly reduced for Lap-DG vs. Op-DG (WMD = −113.6; 95% CrI −129.5; −97.3) and LapAs-DG vs. Op-DG (WMD = −29.2; 95% CrI −40.2; −18,4). No statistically significant difference was observed between Lap-DG and Rob-DG (WMD = 14.49; 95% CrI −37.25; 8.38). Perioperative outcomes, like time to first flatus and time to first oral intake, were reported in seven studies, with 3.372 [28,30,32,33,34,35,36] and 2.605 [29,30,31,33,34,35,36] patients, respectively. No statistically significant differences in time to first flatus were observed for Lap-DG vs. Op-DG (WMD = 0.61; 95% CrI −0.33; 1.53), LapAs-DG vs. Op-DG (WMD = 0.21; 95% CrI −0.46; 0.87), and Rob-DG vs. Op-DG (WMD = 0.91; 95% CrI −0.73; 2.51). Comparable time to first flatus was also observed when comparing relative effects among minimally invasive techniques. Similarly, no statistically significant differences in time to first oral intake were observed, considering relative effects among all treatments. HLOS [28,29,30,31,32,33,34,35,36] was comparable across treatments, with no statistically significant differences between Lap-DG and Op-DG (WMD = 0.9; 95% CrI −0.74; 2.86), LapAs-DG and Op-DG (WMD = 0.67; 95% CrI −1.03; 2.41), Rob-DG and Op-DG (WMD = 1.2; 95% CrI −2.51; 5.22), LapAs-DG and Lap-DG (WMD = 0.23; 95% CrI −2.87; 2.12), and Rob-DG and Lap-DG (WMD = 0.3; 95% CrI −3.13; 3.72). No significant differences were observed for overall complications, reoperation rate, pulmonary complications, postoperative bleeding, SSI, and cardiovascular complications. No significant differences were found in terms of total number of harvested lymph nodes (nine studies, 1961 patients) [28,29,30,32,33,34,35,36,37] and tumor-free resection margins (six studies, 1863 patients). [28,29,30,31,32,34] The league table for all outcomes with both direct and indirect comparisons is reported in Table 3. The sensitivity analysis showed the robustness of the findings.

## 4. Discussion

This study suggests comparable anastomotic leak, overall complications, severe postoperative complications (CD > 3), and in-hospital mortality RR for Op-DG, LapAs-DG, Lap-DG, and Rob-DG for locally AGC. Similarly, conversion to open procedure, reoperation, time to oral intake, and time to ambulation were similar. Notably, despite the longer OT, Lap-DG, LapAs-DG, and Rob-DG were associated with a trend toward improved intraoperative blood loss, time to first flatus, and HLOS. The total number of harvested lymph nodes and tumor-free resection margin seem comparable among techniques.

Despite the efforts made in gastric cancer screening and surveillance, the majority of tumors are detected at an advanced stage (T3/4) at the moment of diagnosis [4]. Locally AGC remains a challenge that requires a comprehensive understanding of its characteristics and the spectrum of potential therapeutic strategies available. In this context, the multidisciplinary approach represents the pivotal point in the management and involves chemotherapy, surgery, and radiotherapy [2]. In recent times, insights into the biological behavior of GCs have significantly influenced treatment protocols and drug choices for neoadjuvant chemotherapy. Current approved chemotherapeutic agents are anti-HER2 drugs such as trastuzumab and anti-angiogenic pathway drugs such as ramucirumab [52,53]. Notably, emerging evidence supports the use of targeted therapy and immunotherapy, with established recommendations and standards for molecular marker testing, including mismatch repair/microsatellite instability (MMR/MSI) and programmed death ligand-1 (PD-L1) [54,55]. Alongside chemotherapeutic agents, the surgical procedure still represents the cornerstone of AGC management [2]. While minimally invasive approaches have demonstrated substantial benefits in terms of rapid postoperative recovery and an acceptable surgical complication rate for early gastric tumors, some authors argue that these techniques can provide similar advantages for the locally AGC treatment. In fact, Op-DG currently remains the gold standard treatment for locally AGC located in the antrum; however, minimally invasive laparoscopic-assisted and totally laparoscopic approaches have gained increasing recognition over the last three decades [23,25,27,56,57,58]. The main aspects of these minimally invasive techniques involve enhanced visualization and precision while minimizing tissue trauma. Nevertheless, certain constraints, such as limited instrument motion range and suboptimal ergonomics, make the procedure technically demanding in achieving anatomical resection and thorough lymphadenectomy. Considering these aspects, the advent of robotic platforms has sparked renewed enthusiasm. This is attributed to several factors, including enhanced ergonomics, greater stability of the surgical field, advanced high-definition 3D visualization, and augmented precision, all coupled with the expanded motion range of instruments [33].

It has been shown that in-hospital mortality after distal gastrectomy for locally AGC is up to 1% [20,27,29,32]. In our network analysis, the estimated mortality rates for Op-DG, LapAs-DG, Lap-DG, and Rob-DG were 0.36%, 0.24%, 0.39%, and 0.0%, respectively. No differences were detected in the quantitative analysis with comparable RRs among treatments; the global related heterogeneity was low (I^2^ = 12.4%), thus adding robustness to the results. These findings are similar to those reported by Park et al. for LapAs-DG vs. Op-DG (0% vs. 1%; *p* = 0.75) [31]. Additionally, Huscher et al. described comparable mortality rates for Lap-DG vs. Op-DG (3.3% vs. 6.7%; *p* = ns) [29]. Notably, caution is recommended because our results may be influenced by potential confounders such as different perioperative protocols, patient selection bias, hospital volume, and learning curves, while the specific cause of death was not specifically reported. The incidence of anastomotic leak after distal gastrectomy for gastric cancer has been previously reported to be up to 2.7% [59]. In the present analysis, Op-DG, LapAs-DG, Lap-DG, and Rob-DG were associated with 1.2%, 1.5%, 1.2%, and 0.0% estimated overall anastomotic leak rate, respectively. Again, the quantitative analysis showed no significant RR differences among treatments with a low global heterogeneity (I2 = 23%). Our results are in line with what was reported by Wang et al., which described comparable anastomotic leak rates for LapAs-DG vs. Op-DG (1.4% vs. 1.8%, *p* = 0.72) [35]. Similarly, comparable anastomotic leak rates were reported by Lee et al. for the Lap-DG vs. Op-DG (1.8% vs. 1.4%, *p* = 0.8) comparison [30]. The overall complication rate has been previously reported to be up to 25% in patients with locally AGC undergoing distal gastrectomy [60]. Notably, the quantitative network assessment did not show significant differences among treatments for both overall complications and severe (CD > 3) complications. Again, cardiovascular complications, pancreatic fistula, pulmonary complications, SSI, conversion to open surgery, and the need for postoperative blood transfusion were comparable among treatments. Interestingly, our analysis showed that Lap-DG, LapAs-DG, and Rob-DG are associated with significantly reduced intraoperative blood loss compared to Op-DG. Small surgical incisions and fine tissue dissections performed in minimally invasive approaches may explain this result. Operative time was longer for LapAs-DG, Lap-DG, and Rob-DG compared to ODG; this is hypothetically related to technical reasons such as challenging suprapancreatic/hepatic hilar lymph nodes and omentum dissection in LapAs-DG and Lap-DG whereas in Rob-DG docking time should be considered [61,62]. Compared to Op-DG, a trend toward improved functional outcomes was noticed for minimally invasive techniques with shorter time to first flatus and HLOS. Given the moderate heterogeneity, our results should be interpreted thoughtfully since patients’ comorbid conditions, ASA grade, BMI, smoking status, antibiotic treatments, the technique for intestinal reconstruction (Billroth I vs. Billroth II vs. Roux-en-Y) [63], lymphadenectomy extent (D1 vs. D1+ vs. D2), omentectomy (total vs. partial vs. non-performed), hospital protocols [64], application of enhanced recovery protocols (ERAS) [28], techniques for alimentary reconstruction (i.e., Roux-en-Y vs. Billroth), surgeons’ experience, and hospital volume could theoretically impact all these short-term outcomes.

D2 lymphadenectomy for gastric cancer is a matter of debate [59,65]. Lymph node metastasis has been shown to be a principal prognostic factor, while adequate lymphadenectomy is essential for accurate pathologic staging. Also, lymphadenectomy reduces the incidence of loco-regional recurrences, thus potentially contributing to better survival [66,67,68,69]. As D2 lymphadenectomy is time-consuming and technically demanding, concerns exist that more extended dissections may correlate with higher morbidity, especially in patients who underwent neoadjuvant treatment [70]. In locally AGC, pancreas and spleen preserving D2 lymphadenectomy is advocated as the gold standard approach; however, its role in survival improvement remains controversial [65]. The 8th AJCC edition endorses at least 15 lymph nodes for a correct and reliable N-staging, while in other studies, more extended dissections seem associated with a survival improvement [71]. In this study, no differences were found among surgical techniques in the total number of harvested lymph nodes. This result is different from the data by Memon et al. that reported a significantly lower number of harvested lymph nodes for LapAs-DG compared to Op-DGDG (*p* = 0.0002) [11]. Hypothetically, Rob-DG would enable improved lymph node dissections and may help surgeons in cases of complex anatomy; however, this benefit needs to be proven [33]. Notably, the global heterogeneity was moderate, suggesting that these results might have been influenced by institutional operative volume, neoadjuvant treatments, surgeons’ experience and motivation, intraoperative labeling of lymph node location, technical constraints, and pathologist experience.

Operating surgeon learning curve and expertise might have impacted patient outcomes and can be a significant source of bias [72,73]. It has been shown that these operator-related factors are of utmost importance for determining operative time, intraoperative blood loss, total number of harvested lymph nodes, and overall complications [74,75]. In the present meta-analysis, some trials failed to describe specific data about operators who performed a distal gastrectomy, while nine trials described the operating surgeons’ proficiency [29,30,31,32,33,34,35,36,37]. In addition, surgical quality control with intraoperative images, videos, and checklist evaluation were described to assess the quality of lymphadenectomy and gastric resection. These data were reported from high-volume teaching hospitals; therefore, they should be interpreted carefully and may not be applicable to small, non-teaching hospitals.

Using Bayesian meta-analytical methods, we were able to globally synthesize data from numerous studies and, therefore, rank the treatments. This study was planned in agreement with the PRISMA 2020 guidelines and followed a pre-defined methodology that was expressed in PROSPERO. This included comprehensive outcome measures and the evaluation of quality at the study level (risk of bias) and confidence in results at the outcome level (CINeMA). The selection criteria led to a homogenous population for some of the primary outcomes, as confirmed by low heterogeneity. Further, with the network methodology, we were able to assess indirect comparisons that have never been reported previously. This study has several limitations. First, although the transitivity assumption was met with no evidence of statistically significant inconsistency in the network analysis, the accuracy of our results can be tempered by differences in neoadjuvant/adjuvant treatments with a presumed effect on postoperative complications. In this study, only a small portion of patients underwent preoperative neoadjuvant treatment. Therefore, further evaluations are necessary to deeply assess short-term outcomes and survival after neoadjuvant treatment. Second, even though only RCTs were included in our analyses, the quality of evidence remained moderate, in part due to no blinding of patients and/or surgeons, limited power in some trials, different methods for randomization, and quality control. Third, despite the focus analysis on locally AGC, the pathological assessment downgraded the clinical staging to early gastric cancer. Fourth, all studies were performed in Eastern countries hence results might not be generalizable to Western countries/patients. Fifth, surgeries were performed by expert surgeons in high-volume referral centers; therefore, the results may not be generalizable.

## 5. Conclusions

In the setting of locally AGC, minimally invasive LapAs-DG, Lap-DG, and Rob-DG performed in referral centers by dedicated surgeons seem associated with comparable short-term outcomes compared to Op-DG. Future studies are mandatory to corroborate this preliminary evidence, especially in the setting of Lap-DG and Rob-DG.

## Figures and Tables

**Table 1:** Characteristics of patients undergoing open (Op-DG), laparoscopic (Lap-DG), laparoscopic-assisted (LapAs-DG), and robotic distal gastrectomy (Rob-DG). Int, intestinal; Diff, differentiated; Und, undifferentiated; Neoadj, neoadjuvant treatment; Adj, adjuvant treatment; NR, not reported; AJCC, American Joint Committee on Cancer. Data are reported as numbers, mean ± standard deviation, and median (range). NR not reported.

Author, Year Country	Period	Surgical Approach	No. Pts	Age (yrs)	Gender M/F	BMI (kg/m^2^)	Staging System	Stage Ia	Stage Ib	Stage II	Stage III	Stage IV	Tumor Histology Int/Diff	Tumor Histology Diffused/Und	Neoadj/ Adj
Huscher, 2005 Italy [29]	1992–1996	Op-DG	29	63.6 ± 13.2	21/8	NR	AJCC 1997	6	3	5	11	4	18	11	NR
		Lap-DG	30	63.2 ± 12.5	18/12			7	6	4	8	5	16	14	
Jin chen Hu, 2012 China [28]	2009–2011	Op-DG	20	64.5 ± 6.5	12/8	23.4 ± 2.6	NR	1		6	11	2	NR	NR	0/NR
		Lap-DG	22	62.5 ± 6.75	10/12	22.9 ± 2.2		1		10	10	1			0/NR
Hu Y, 2016 China [36]	2012–2014	Op-DG	520	55.8 ± 11.1	346/174	22.7 ± 3.2	AJCC 7th	99	53	138	221	8	365	155	0/NR
		Lap-DG	519	56.5 ± 10.4	380/139	22.7 ± 3.3		87	64	77	219	11	361	158	0/NR
Shi, 2018 China [37]	2010–2012	Op-DG	102	NR	NR	NR	AJCC 6th	0	10	NR	NR	NR	38	122	0/NR
		Lap-DG	94					0	16				45	117	0/NR
Park, 2018 Korea [31]	2010–2011	Op-DG	96	60.1 ± 8.2	65/31	23.3 ± 3.1	AJCC 7th	22	14	33	23	4	96	0	0/NR
		Lap-DG	100	58.6 ± 8.9	69/31	23.7 ± 3.0		27	15	29	28	1	100	0	0/NR
Wang, 2019 China [35]	2014–2017	Op-DG	220	60.6 ± 10.2	133/87	23.5 ± 3.3	AJCC 7th	41	27	63	83	6	59	161	0/NR
		LapAs-DG	222	59.4 ± 12.4	144/78	23.1 ± 3.1		44	31	63	80	4	47	175	0/NR
Li, 2019 China [34]	2015–2017	Op-DG	50	61 ± 2.25	34/16	22.6 ± 0.9	AJCC 7th	10	6	19	12	0	10	40	50/50
		LapAs-DG	45	59 ± 3.25	32/13	23.5 ± 1		7	6	18	10	0	10	35	45/45
Lee, 2019 Korea [30]	2011–2015	Op-DG	498	59.6 ± 11.5	346/152	23.7 ± 3.3	NR	167		170	154	7	498	0	0/NR
		Lap-DG	513	59.8 ± 11.1	370/143	23.5 ± 2.9		181		151	172	9	513	0	0/NR
Lu, 2021 China [33]	2017–2020	Lap-DG	142	59.3 ± 11.3	90/52	22.7 ± 3.3	AJCC 8th	43		36	63	0	56	86	0/19
		Rob-DG	141	59.4 ± 10.2	94/47	23.2 ± 3		55		33	53	0	52	89	0/18
Etoh 2023 Japan [32]	2009–2016	Op-DG	233	66 ± 7.8	160/73	22.7	AJCC 7th	45		50	67	71	0	NR	0/NR
		Lap-DG	227	64 ± 6.6	156/71	22.3		52		39	64	72	0		0/NR

**Table 2:** Descriptive statistics stratified according to different treatments. Open (Op-DG), laparoscopic-assisted (LapAs-DG), totally laparoscopic (Lap-DG), and robotic distal gastrectomy (Rob-DG). SSI, surgical-site infection; HLOS, hospital length of stay. Values are presented as percentages for categorical variables and as mean (range) for continuous variables.

Op-DG	LapAs-DG	Lap-DG	Rob-DG	
1.2 (0.0–2.2)	1.5 (0.0–2.2)	1.2 (0.0–1.7)	0.0 (0.0–0.0)	Anastomotic leak
6.4 (0.0–17.7)	3.8 (2.2–13.3)	6.4 (0.0–11)	1.4 (1.4–1.4)	Clavien–Dindo III–IV
18.4 (10.7–46)	15 (11.1–36.2)	16 (11–30.2)	9 (9–9)	Overall complication
0.3 (0.0–6.8)	0.2 (0.0–0.4)	0.03 (0.0–3.3)	0.0 (0.0–0.0)	In-hospital mortality
2.8 (0.0–6.8)	1.1 (0.0–1.3)	2.9 (0.0–6.6)	1 (1–1)	SSI
2 (0.0–6.1)	1 (0.0–2.3)	1.1 (0.0–2.1)	1 (1–1)	Bleeding requiring transfusion
4.1 (0.0–21)	5 (2.1–7.3)	4.7 (2.6–17.2)	5.9 (5.9–5.9)	Pulmonary complications
0.0 (0.0–3.1)	1.1 (0.0–4.2)	0.0 (0.0–1.4)	1.1 (1.1–1.1)	Cardiovascular complications
1.8 (0.0–3.6)	1.8 (0.0–2.1)	1.1 (0.0–1.7)	0.0 (0.0–0.0)	Need for reoperation
1.9 (0.0–8.2)	0.9 (0.0–2.1)	1.1 (0.0–2.1)	NR	Positive resection margins (R1)
180.8 (123.2–209.9)	231.3 (196.0–227.1)	240 (185–240)	201 (201–201)	Operative time (minutes)
165 (58.7–523)	139 (95–236)	127 (84–320)	41.2 (41.2–41.2)	Intraoperative blood loss (mL)
3.7 (2.2–4.0)	3.1 (2.5–3.5)	3.2 (2.3–3.4)	3.2 (3.2–3.2)	Time to first flatus (days)
4.5 (2.9–7.8)	4.6 (3.4–6.5)	4.4 (3.2–6.1)	3.5 (3.5–3.5)	Time to oral intake (days)
10.6 (8.1–18.8)	9.5 (8.8–10.7)	9.1 (8.4–11.2)	7.9 (7.9–7.9)	HLOS (days)
40.2 (31.4–76.5)	38.5 (30.0–46.6)	37.1 (29.1–47.2)	40.9 (40.9–40.9)	Total No. lymph nodes

**Table 3:** League table. Each row represents a specific outcome. Values in each column represent the relative effect of the referral treatment (bold) with the comparator. Values are expressed as risk ratio (RR) and 95% credible intervals (95% CrIs). I^2^: heterogeneity.

				I^2^ (95% CrI)	Outcomes
**LapAs-DG**	0.79 (0.28–2.22)	0.63 (0.29–1.39)	0.67 (0.19–2.50)	23.1	Anastomotic leak
1.27 (0.45–3.53)	**Lap-DG**	0.81 (0.38–1.72)	0.86 (0.39–1.86)		
1.58 (0.72–3.39)	1.24 (0.58–2.61)	**Op-DG**	1.062 (0.37–3.06)		
1.49 (3.99–5.40)	1.17 (0.54–2.54)	0.94 (0.32–2.72)	**Rob-DG**		
**LapAs-DG**	0.64 (0.32–1.58)	1.55 (0.33–1.11)	0.44 (0.16–1.61)	0.0	Clavien–Dindo III–IV
1.56 (0.63–3.15)	**Lap-DG**	0.85 (0.50–1.46)	0.69 (0.34–1.52)		
1.82 (0.90–3.07)	1.17 (0.69–1.99)	**Op-DG**	0.80 (0.34–2.14)		
2.26 (0.62–6.25)	1.45 (0.66–2.96)	1.25 (0.47–2.90)	**Rob-DG**		
**LapAs-DG**	0,98 (0.63–1.62)	1.02 (0.75–1.40)	0.65 (0.32–1.48)	45.5	Overall complications
1.02 (0.62–1.58)	**Lap-DG**	1.03 (0.72–1.42)	0.66 (0.38–1.22)		
0.98 (0.71–1.32)	0.97 (0.70–1.39)	**Op-DG**	0.64 (0.34–1.34)		
1.54 (0.68–3.09)	1.51 (0.82–2.63)	1.57 (0.75–2.94)	**Rob-DG**		
**LapAs-DG**	0.86 (0.30–2.49)	0.69 (0.32–1.50)	0.78 (0.21–2.89)	12.4	In-hospital mortality
1.16 (0.40–3.36)	**Lap-DG**	0.80 (0.38–1.72)	0.90 (0.41–1.96)		
1.46 (0.67–3.17)	1.25 (0.58–2.67)	**Op-DG**	1.13 (0.38–3.30)		
1.29 (0.35–4.81)	1.11 (0.51–2.42)	0.89 (0.30–2.63)	**Rob-DG**		
**LapAs-DG**	0.84 (0.33–2.22)	0.60 (0.30–1.31)	0.67 (0.20–2.43)	0.0	Postoperative bleeding
1.19 (0.45–3.02)	**Lap-DG**	0.71 (0.35–1.52)	0.80 (0.37–1.76)		
1.67 (0.77–3.29)	1.41 (0.66–2.82)	**Op-DG**	1.12 (0.40–3.09)		
1.49 (0.41–4.96)	1.25 (0.57–2.73)	0.89 (0.32–2.47)	**Rob-DG**		
**LapAs-DG**	0.88 (0.31–2.51)	0.68 (0.31–1.48)	-	60.2	Pancreatic injury/leak
1.13 (0.40–3.23)	**Lap-DG**	0.766 (0.35–1.67)	-		
1.48 (0.67–3.22)	1.31 (0.60–2.81)	**Op-DG**	-		
-	-	-	**Rob-DG**		
**LapAs-DG**	0.83 (0.33–2.18)	0.63 (0.32–1.35)	0.66 (1.97–2.35)	11.3	SSI
1.21 (0.46–3.02)	**Lap-DG**	0.76 (0.39–1.56)	0.79 (0.36–1.72)		
1.59 (0.74–3.14)	1.32 (0.64–2.56)	**Op-DG**	1.04 (0.38–2.84)		
1.52 (0.43–5.08)	1.26 (0.58–2.71)	0.96 (0.35–2.60)	**Rob-DG**		
**LapAs-DG**	0.90 (0.41–1.94)	0.60 (0.37–1.11)	0.67 (0.25–2.05)	23.4	Pulmonary complications
1.11 (0.51–2.43)	**Lap-DG**	0.66 (0.38–1.30)	0.75 (0.38–1.60)		
1.68 (0.90–2.73)	1.51 (0.77–2.61)	**Op-DG**	1.12 (0.47–2.77)		
1.49 (0.48–4.01)	1.34 (0.62–2.64)	0.89 (0.36–2.14)	**Rob-DG**		
**LapAs-DG**	0.79 (0.27–2.32)	0.68 (0.31–1.49)	0.73 (0.19–2.75)	26.4	Cardiovascular complications
1.26 (0.43–3.66)	**Lap-DG**	0.86 (0.40–1.86)	0.92 (0.42–1.99)		
1.47 (0.67–3.18)	1.17 (0.54–2.51)	**Op-DG**	1.07 (0.36–3.15)		
1.37 (0.36–5.15)	1.09 (0.50–2.36)	0.93 (0.32–2.76)	**Rob-DG**		
**LapAs-DG**	0.85 (0.30–2.46)	0.75 (0.35–1.60)	0.73 (0.20–2.76)	69.3	Reoperation
1.18 (0.41–3.38)	**Lap-DG**	0.88 (0.41–1.92	0.86 (0.40–1.87)		
1.33 (0.62–2.78)	1.13 (0.52–2.46)	**Op-DG**	0.97 (0.33–2.89)		
1.37 (0.36–5.08)	1.16 (0.53–2.52)	1.03 (0.35–3.04)	**Rob-DG**		
**LapAs-DG**	1.23 (0.45–3.51)	0.82 (0.39–1.76)	-	0.0	R1/R2
0.77 (0.28–2.21)	**Lap-DG**	0.64 (0.30–1.42)	-		
1.23 (0.57–2.57)	1.57 (0.70–3.36)	**Op-DG**	-		
-	-	-	**Rob-DG**		
				I^2^ (95% CrI)	
**LapAs-DG**	−84.4 (−103.7; −64.8)	29.17 (18.4; 40.0)	−98.9 (−128.7; −68.7)	68.1	Intraoperative blood loss (mL)
84.41 (64.8; 103.7)	**Lap-DG**	113.6 (97.3; 129.5)	−14.5 (−37.3; 8.4)		
−29.17 (−40.0; −18.4)	−113.6 (−129.5; −97.3)	**Op-DG**	−128.1 (−155.9; −100.0)		
98.9 (68.7; 128.7)	14.5 (−8.4; 37.3)	128.1 (100.0; 155.9)	**Rob-DG**		
**LapAs-DG**	24.9 (10.2; 39.6)	−39.3 (−49.2; −29.3)	44.6 (18.9; 70.1)	55.2	Operative time (minutes)
−24.9 (−39.6; −10.2)	**Lap-DG**	−64.2 (−75.0; −53.3)	19.6 (−1.3; 40.6)		
39.3 (29.3; 49.2)	64.2 (53.3; 75.0)	**Op-DG**	83.8 (60.2; 107.3)		
−44.6 (−70.1; −18.9)	−19.6 (−40.6; 1.3)	−83.8 (−107.3; −60.2)	**Rob-DG**		
**LapAs-DG**	−0.4 (−1.5; 0.8)	0.2 (−0.5; 0.9)	−0.7 (−2.4; 1.1)	45.7	Time to first flatus (days)
0.39 (−0.8; 1.5)	**Lap-DG**	0.6 (−0.3; 1.5)	−0.3 (−1.6; 1.0)		
−0.2 (−0.9; 0.5)	−0.6 (−1.5; 0.3)	**Op-DG**	−0.9 (−2.5; 0.7)		
0.7 (−1.1; 2.4)	0.3 (−1.0; 1.6)	0.9 (−0.7; 2.5)	**Rob-DG**		
**LapAs-DG**	−0.3 (−5.0; 4.3)	0.4 (−3.1; 4.0)	−0.7 (−7.5; 6.1)	67.1	Time to oral intake (days)
0.3 (−4.3; 5.0)	**Lap-DG**	0.8 (−2.1; 3.7)	−0.4 (−5.4; 4.6)		
−0.4 (−4.0; 3.1)	−0.8 (−3.7; 2.1)	**Op-DG**	−1.2 (−7.0; 4.6)		
0.7 (−6.1; 7.5)	0.4 (−4.6; 5.4)	1.2 (−4.6; 7.0)	**Rob-DG**		
**LapAs-DG**	−0.2 (−2.9; 2.1)	0.7 (−1.0; 2.4)	−0.5 (−4.9; 3.6)	54.7	HLOS (days)
0.2 (−2.1; 2.9)	**Lap-DG**	0.9 (−0.7; 2.9)	−0.3 (−3.7; 3.1)		
−0.7 (−2.4; 1.0)	−0.9 (−2.8; 0.7)	**Op-DG**	−1.2 (−5.2; 2.5)		
0.5 (−3.6; 4.9)	0.3 (−3.1; 3.7)	1.2 (−2.5; 5.2)	**Rob-DG**		
**LapAs-DG**	−1.5 (−9.7; 5.9)	−0,5 (−5.4; 3.9)	−0.5 (−13.9; 12.3)	6.5	Total No. lymph nodes
1.5 (−5.9; 9.7)	**Lap-DG**	0.9 (−5.1; 7.5)	1.0 (−9.5; 11.5)		
−0.5 (−3.9; 5.4)	−0.9 (−7.5; 5.2)	**Op-DG**	0.0 (−12.4; 12.1)		
0.5 (−12.3; 13.9)	−1.0 (−11.5; 9.5)	−0.0 (−12.1; 12.4)	**Rob-DG**		

## Data Availability

Data generated at a central, large-scale facility are available upon request from the corresponding author.

## Supplementary Materials

The following supporting information can be downloaded at https://www.mdpi.com/article/10.3390/cancers16091620/s1. Figure S1: Risk of bias for randomized controlled trials (RCTs) was assessed using the Cochrane risk-of-bias tool. Green circle: low risk of bias. Red circle: high risk of bias. Yellow circle: unclear risk of bias. Table S1: Quality of the included RCTs. File S1—PRISMA checklist.

## Abbreviations

Open distal gastrectomy (Op-DG), advanced gastric cancer (AGC), laparoscopic-assisted distal gastrectomy (LapAs-DG), early gastric cancer (EGC), laparoscopic distal gastrectomy (Lap-DG), robotic distal gastrectomy (Rob-DG), randomized controlled trials (RCTs), body mass index (BMI), surgical-site infection (SSI), hospital length of stay (HLOS), risk ratio (RR), weighted mean difference (WMD), intestinal (Int), differentiated (Diff), undifferentiated (Und), neoadjuvant treatment (Neoadj), adjuvant treatment (Adj), American Joint Committee on Cancer (AJCC).
