# Supplementary material for: Short-Term Outcomes Analysis Comparing Open, Laparoscopic, Laparoscopic-Assisted, and Robotic Distal Gastrectomy for Locally Advanced Gastric Cancer: A Randomized Trials Network Analysis
Source: Cancers (Basel). 2024 Apr 23;16(9):1620. doi: 10.3390/cancers16091620 (PMC11083793; doi:10.3390/cancers16091620)
Supplement: Supplementary file 1 [file cancers-16-01620-s001.zip › Suppl Table S1.pdf]

**Author, year *country***	**Study design**	**Randomization method**	**Surgeons' eligibility**	**Surgical quality control**	**Blinding**	**Power analysis**
Huscher, 2005 *Italy*	nr	nr	Single experienced surgeon	nr	no	nr
Jin chen Hu, 2012 *China*	nr	nr	nr	nr	P: blind. S: not blind. Stat: blind	Y
Yanfeng Hu, 2016 *China*	non-inferiority RCT	Computer-based stratified (institution, age, histology preoperative TNM)	>50 ODG and >50 LADG<br>>300 gastrectomies/year per institution, qualified surgeons based on National academic committee	Video review and photographic documentation resection margins, abdominal incisions, lymphadenectomy	no	Y
Shi, 2018 *China*	non-inferiority RCT	Block randomization<br>(fixed block size=ABBA)	>50 LADG<br>>50 ODG	Video review and photographic documentation	no	Y
Park, 2018 *Korea*	nr	Web-based randomization system with fixed block size 1:1 randomization	>30 LADG	Surgical checklist evaluation	no	Y
Wang, 2019 *China*	non-inferiority RCT	Computer-based non-stratified 1:1 randomization	>60 LADG<br>>60 ODG	Video review and photographic documentation resection margins, abdominal incisions, lymphadenectomy	no	Y
Li, 2019 *China*	non-inferiority RCT	Computer-based stratified 1:1 randomization	>600 total LADG<br>annual surgical volume of approximately 300 LADG/ODG	nr	no	nr
Lee, 2019 *Korea*	non-inferiority RCT	Stratified 1:1 randomization using a confidential block size	>50 LADG<br>>50 ODG	Video review and photographic documentation resection margins, abdominal incisions, lymphadenectomy	no	Y
Lu, 2021 *China*	non-inferiority RCT	Computer-based stratified 1:1 (LDG vs. RDG) ratio	>300 LADG<br>>50 RDG	Video review and photographic documentation resection margins, abdominal incisions, lymphadenectomy	no	Y
Etoh, 2023 *Japan*	non-inferiority RCT	Computer-based stratified (histology preoperative TNM, institution)	> 50 ODG and > 20 gastrectomies/year per institution, qualified surgeons according to Endoscopic Surgical Skill Qualification System (JSES)	Photographic documentation review	no	nr

Supplementary Table S1. Randomized Clinical Trials (RCTs) quality evaluation. Open Distal Gastrectomy (**ODG**), Laparoscopic Distal Gastrectomy (**LDG**), Laparoscopic-Assisted Distal Gastrectomy (**LADG**), and Robotic Distal Gastrectomy (**RDG**). P patient, S surgeon, Stat statistician, Y yes, UP under-powered, JSES Japan Society for Endoscopic Surgery.
