# Supplementary figures and images for: Short-Term Outcomes Analysis Comparing Open, Laparoscopic, Laparoscopic-Assisted, and Robotic Distal Gastrectomy for Locally Advanced Gastric Cancer: A Randomized Trials Network Analysis
Source: Cancers (Basel). 2024 Apr 23;16(9):1620. doi: 10.3390/cancers16091620 (PMC11083793; doi:10.3390/cancers16091620)

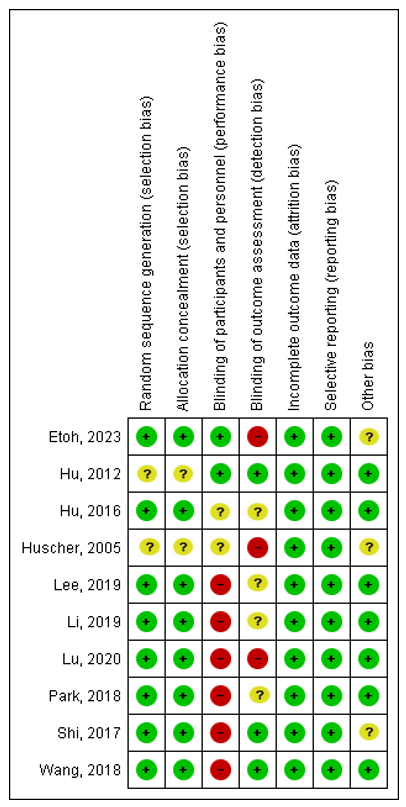

Supplement: Supplementary file 1 [file cancers-16-01620-s001.zip › Suppl Figure S1 .tiff]
